# Speed of flea knockdown of spinosad compared to afoxolaner, and of spinosad through 28 days post-treatment in controlled laboratory studies

**DOI:** 10.1186/s13071-015-1195-5

**Published:** 2015-11-09

**Authors:** Daniel E. Snyder, Anthony J. Rumschlag, Lisa Marie Young, William G. Ryan

**Affiliations:** Elanco Animal Health, Greenfield, IN USA; Ryan Mitchell Associates LLC, Westfield, NJ USA

**Keywords:** Spinosad, Afoxolaner, Flea, *Ctenocephalides felis*, Efficacy, Speed of kill

## Abstract

**Background:**

The speed of flea knockdown by different products and their duration of effectiveness are factors which affect veterinarian prescribing decisions. To further validate the month-long pulicidal effectiveness of spinosad and determine its rate of flea knockdown to that of afoxolaner, three studies were conducted in two laboratories in the United States, utilizing flea infestations from colonies which are regularly refreshed through introduction of locally caught fleas.

**Methods:**

All study assessors were blinded, dogs were ranked by pre-study flea counts and randomized accordingly, and treatments administered on Day 0. All studies included a negative control group; two also included an afoxolaner group. In one study, flea challenges for treated and control dogs (10 per group) were completed 21 and 28 days after treatment and counts were performed 24 h later. In each of two speed-of-knockdown (SOK) studies, 36 dogs were randomized, six dogs per group, to: untreated controls; administered oral afoxolaner (2.6-6.2 mg/kg); or oral spinosad (32.1-59.2 mg/kg). In the SOK studies, live fleas from Day -1 infestations were counted after being combed off at 1 and 3 h after treatment, and after reinfestations on Day 7.

**Results:**

There were no treatment-related adverse events. Spinosad was 98.6 % effective at 28 days post treatment. For SOK, geometric mean live flea counts for afoxolaner were not different from controls at any assessment. For spinosad, all mean counts were significantly lower than in controls (*p* ≤ 0.0128) except at 1 h post treatment in both studies. Spinosad was significantly more effective than afoxolaner in both studies at 3 h post treatment (*p* ≤ 0.0065) and post-Day 7 infestation (*p* ≤ 0.0054), and at 1 h post treatment (*p* = 0.0276) and post-Day 7 infestation in one study.

**Conclusions:**

These data validate spinosad’s faster onset of flea knockdown than afoxolaner against infestations present at the time of treatment, and faster residual speed of flea knockdown for at least 7 days post treatment, and confirm spinosad’s extended residual speed of kill for at least 28 days post treatment.

## Background

The widespread establishment of monthly treatments to control canine parasites began in the 1980s and early 1990s with the release of the monthly orally administered macrocyclic lactone products that produced a major advance in the prevention of heartworm disease. These treatments also offered effectiveness against intestinal nematode parasites. Subsequent to the emergence of these products, monthly flea control became accepted in the 1990s with the release of the low volume topical flea control products containing imidacloprid or fipronil. The monthly oral insect growth regulator (IGR) lufenuron, which could prevent the hatching of viable larvae from flea eggs was registered in 1994. A limitation of this, and IGRs in general, is the absence of adult flea effectiveness, and perhaps for this reason the topical flea adulticide knockdown products dominated the market throughout the remainder of the decade and beyond. However, the registration in 2000 of nitenpyram highlighted the potential for oral treatments. Nitenpyram provided a very rapid onset of flea knockdown, and was demonstrated to kill more than 99 % of fleas within four to six hours of treatment. Additionally, studies with nitenpyram demonstrated that systemically acting pulicides could provide a faster onset of action against fleas than topically applied products in dogs and in cats [1–3]. The major limitation of nitenpyram was its ultra short duration of action − little more than a day. There was therefore a need for an oral product that would quickly eliminate existing flea burdens and maintain flea killing activity for a month, thereby aligning with the well-established paradigm of monthly parasite control.

Spinosad was the first orally administered flea control product to provide one month of residual adult flea knockdown activity. With 100 % effectiveness reported within 4 h of treatment of existing infestations, spinosad was shown to have a comparable onset of activity to nitenpyram, but also provided a duration of flea knockdown that reduced flea egg output over one month by >99.8 % [4]. Controlled field studies with spinosad reported from 2007 through 2015 have confirmed its ongoing and consistent efficacy in field studies, achieving a high level of flea control in households that included treated dogs and cats, and alleviating the clinical signs of flea infestation, including pruritus [5–10].

In recent years, a number of reports of purpose-designed studies have suggested the flea activity of spinosad to be inconsistent under laboratory conditions. These studies may not be reflective of real world conditions because they have used challenges with selected flea isolates such as the Kansas 1 (KS1) strain [11, 12], maintained under laboratory conditions since 1990, or laboratory-reared fleas from Europe that were transported internationally for testing in various contract testing laboratories [13, 14].

To further validate the month-long pulicidal effectiveness of spinosad, and to determine the relative rate of flea knockdown of spinosad to that of afoxolaner, the laboratory studies reported in this paper were undertaken using fleas from colonies in which genetics are regularly modified by the introduction of locally sourced wild-type fleas (*Ctenocephalidesfelis*) to reflect potentially changing field challenges. Afoxolaner is the second orally administered systemically acting compound to be labeled for one month of flea effectiveness. As earlier work had demonstrated that spinosad was 100 % effective in four hours, the studies reported herein compared the products at up to 3 h post treatment or post-infestation on Day 7 [4].

## Methods

### Ethical approval

The studies summarized in this report were performed at 2 independent laboratories in accordance with Good Clinical Practices as described in VICH guideline GL9, Good Clinical Practice (June 2000). All protocols were reviewed and approved by the respective Institutional Animal Care and Use Committee. One study utilized a randomized block, blinded, parallel-arm, negative control design and two studies also included a positive controlled group treated with afoxolaner. Individual dogs were considered the experimental unit.

Flea infestations were completed using each laboratory’s established in-house *C. felis* colonies to which recent isolates obtained from a local source had been introduced within 12 months of the beginning of each study. Approximately 100 unfed adult fleas were counted from the colony into application vials and then deposited along the dorsal mid-line, from the shoulder to hip, of each dog. Each infested dog was then hand-restrained within its primary housing run for sufficient time to allow parasites to penetrate the hair coat. Disposable gloves and aprons were worn and changed between each of the treatment groups.

Staff performing the post-treatment flea comb counts and making individual animal observations remained blinded to the actual treatment groups. At the assigned time points, fleas were removed and counted by thoroughly combing animals with fine-tooth flea combs. Flea counts were completed using each facility’s standard operating protocol of thorough whole body combing of each dog for at least 10 min, regularly removing fleas and hair from the comb, determining whether or not fleas were dead, showing abnormal movement (moribund but counted as live), or live and apparently normal. Only live fleas were counted and recorded, and any flea movement was recorded as normal or abnormal. Fleas were discarded after removal from a dog. Beyond the initial 10 min combing, if additional fleas were found combing was continued for an additional five minutes until no additional fleas were found. The fleas and hair were disposed of in containers of a soap solution. To avoid cross contamination, each treatment group had new separate comb pairs assigned to it. The group-assigned combs were rinsed with alcohol and wiped clean between each animal. Disposable gloves and aprons were worn and changed between each of the treatment groups.

#### Experimental animals

A total of 92 purebred and mixed-breed dogs, at least 6 months old and weighing between 6.8 and 20.9 kg were used in these studies. All dogs were owned by the respective trial facility and had been present in the respective study facility for at least one week and so were well acclimated. Each dog was uniquely identified by ear tattoo or by a subcutaneous microchip. No medications or vaccinations had been given during the week prior to initial study procedures. Water was provided *ad libitum*. Dogs were fed according to the standard operating procedure of each facility and housed in cages of appropriate size that conformed to accepted guidelines for animal welfare. To be eligible for inclusion in a study, each dog was required to be healthy; to have retained at least 50 fleas from a pre-study infestation of approximately 100 fleas applied 24 h earlier; to not have been exposed to any oral or topical insecticide or IGRs within 100 days of being selected for each study; and to not be used for breeding.

#### Design of studies

##### Efficacy at days 22 and 29

In this study, 24 dogs were ranked on the basis of pre-study flea counts, and the four dogs with the lowest flea burdens were eliminated. The remaining 20 were randomized on the basis of the pre-study counts to either of 2 groups, 10 dogs per group: an untreated control group or a treated group, to receive spinosad (Comfortis®, Elanco; minimum dose 30 mg/kg, United States label dose) according to label recommendations. Dogs were returned to their kennels and flea challenges were completed on Days 21 and 28. Flea counts were completed 24 h after each infestation.

### Spinosad/afoxolaner speed of flea knockdown studies

Studies were undertaken at two separate sites to determine the speed of flea knockdown of spinosad (Comfortis) and afoxolaner (NexGard®, Merial) against infestations present at the time of treatment, and against flea challenge one week after treatment. At one site 57 dogs were screened, 42 dogs at the second site. At each site, the 36 dogs with the highest flea counts were included – the six with the highest flea counts constituted Block 1; the next 6 Block 2, etc., until 6 blocks were formed. Each dog from each block was then randomly assigned to one of six groups until the 36 dogs were assigned to groups (Table 1).

**Table 1:** Treatment groups and description of treatments and flea counts^a^

Group	Dose Rate (per label) (all dogs in fed state)	
A	Spinosad, minimum dose 30 mg/kg	1 hour after treatment (Day 0) and post infestation (Day 7)
B	Spinosad, minimum dose 30 mg/kg	3 hours after treatment (Day 0) and post infestation (Day 7)
C	Afoxolaner minimum dose 2.5 mg/kg	1 hour after treatment (Day 0) and post infestation (Day 7)
D	Afoxolaner minimum dose 2.5 mg/kg	3 hours after treatment(Day 0) and post infestation (Day 7)
E	No treatment administered	
F	No treatment administered	

^a^All dogs were offered their daily ration within the one hour prior to treatment

Treatments were offered free choice in the palm of a gloved right hand (the same type of glove for each dog; a new glove for each dog). Treatments were recorded as “Not accepted” if a dog accepted the product but then ejected it unchewed or partially chewed, or if the dog had not accepted the product free choice within 2 min of it being offered. If the dog did not fully consume the product within 2 min of it being offered, the dog was pilled and recorded accordingly. All treatments were commercially purchased and administered according to label on Day 0 (spinosad minimum dose 30 mg/kg; afoxolaner minimum dose 2.5 mg/kg).

Flea infestations on Day -1 were used to determine the immediate knockdown and speed-of-kill of adult fleas from the treatment administered on Study Day 0. Speed-of-kill efficacy was also assessed on Study Day 7, based on flea infestations made on that study day. The number of live fleas combed off appropriate dogs was counted at 1 and 3 h after dosing on Study Day 0 and after infestation on Study Day 7.

#### Assessment of effectiveness

In the study of efficacy at 22 and 29 days, the primary hypothesis to be tested was:

- H_0_: There will be no effect of spinosad on flea mortality compared to control dogs.
- H_a_: Spinosad will provide significant reductions in live flea counts, compared to controls.

In the speed of kill studies the primary hypothesis was:

- H_0_: There will be no difference between groups in the speed of flea knockdown.
- H_a_: Spinosad will provide significantly faster flea knockdown than afoxolaner.

Descriptive statistics included geometric and arithmetic mean numbers of live fleas (defined as: 1. fleas showing normal movement; and, 2. fleas showing normal movement plus moribund fleas as demonstrated by abnormal movement). To calculate the percent efficacy at each time point, the mean numbers of fleas on dogs in the untreated control group were used for comparison to the flea counts on dogs treated with the study products.

For calculation of geometric means by treatment group at each time point, flea counts were transformed to the natural logarithm of (count + 1) to account for zero values, then back transformed to provide the final value. Percentage efficacy of each treated group with respect to the corresponding control group was calculated using the formula below for each time point.

$$ \%\kern0.5em  Efficacy\kern0.5em =\kern0.5em \frac{Mean\kern0.5em  live\kern0.5em  flea\kern0.5em  count\kern0.5em for\kern0.5em  control\kern0.5em  group- Mean\kern0.5em  live\kern0.5em  flea\kern0.5em  count\kern0.5em for\kern0.5em  treated\kern0.5em  group}{Mean\kern0.5em  live\kern0.5em  flea\kern0.5em  count\kern0.5em for\kern0.5em  control\kern0.5em  group}\kern0.5em x\kern0.5em 100 $$

The log counts of the treated groups were compared to the log counts of the untreated control group using an F-test adjusted for the allocation blocks used to randomize the animals to the treatment groups at each time point separately. Separate calculations were completed for the number of live fleas (Normal + Abnormal) and for the number of live Normal fleas. Statistical comparisons were completed of the percentage reduction from control counts achieved at each time point by the spinosad and afoxolaner treatments. All tests of significance were performed at alpha = 0.05, 2-sided.

## Results

There were no treatment-related adverse events observed in any of the studies.

### Efficacy at 22 and 29 days

The dose of spinosad administered to dogs ranged from 30.3 to 57.6 mg/kg (mean 45.0 mg/kg). For the counts undertaken 24 h after the Day 21 infestation, there were no normal fleas identified in the spinosad-treated dogs. However, dead fleas were mistakenly included in abnormal flea counts, thereby invalidating the total counts as a means of assessing overall effectiveness. At 24 h after the Day 28 infestation, geometric mean counts of live fleas (showing normal movement plus abnormal, or moribund fleas) completed 24 h after the Day 28 infestation were 89.4 in the control group (arithmetic mean 90.6), significantly greater (*p* < 0.0001) than in the spinosad group, 1.28 (arithmetic mean 4.3) for a percentage reduction of 98.6 % (95.3 % based on arithmetic mean) (Table 2). In the spinosad group, counts of moribund fleas in four dogs were 3, 3, 5 and 7; no moribund fleas were found in control dogs. Based on counts of elimination of live fleas with normal movement, spinosad effectiveness on Day 22 was 100 % and on Day 29 was 99.6 % (geometric means).

**Table 2:** Day 29 mean (standard error) (SE) live flea counts^a^ and percentage efficacy of spinosad

	Control	Spinosad
Geometric mean (SE)	89.4 (0.1)	1.3 (0.5)
% Efficacy	-	98.6 %^b^
Arithmetic mean (SD)	90.6 (14.1)	4.30 (9.3)
% Efficacy	-	95.3 %^a^

^a^Moribund fleas counted as live. ^b^Different from control: *p* < 0.0001

#### Speed of kill studies

In both studies combined, 22 of 24 spinosad group dogs and 21 of 24 afoxolaner group dogs did not voluntarily accept treatment and required pilling of the allocated product. The dose rates for afoxolaner ranged from 2.6 to 6.2 mg/kg (means 4.0 mg/kg and 4.7 mg/kg) and of spinosad from 32.1 to 59.2 mg/kg (means 45.1 and 49.1 mg/kg).

**Fig. 1:**
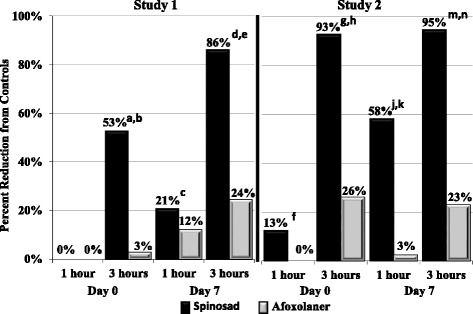
Percent effectiveness, 1 and 3 h post treatment (Day 0) and post infestation (Day 7).  Spinosad,  Afoxolaner. Percentages based on geometric mean live flea counts (normal fleas including those showing abnormal movement) of each group compared to an untreated control group. Different from control: ^a^ *p* = 0.0051; ^c^ *p* = 0.0128; ^d^ *p* = 0.0020; ^g^ *p* = 0.0007; ^j^ *p* = 0.0002; ^m^ *p* = 0.0006. Different from afoxolaner: ^b^ *p* = 0.0065; ^e^ *p* = 0.0054; ^f^ *p* = 0.0276; ^h^ *p* = 0015; ^k^ *p* = 0.0002; ^n^ *p* = 0.0015

**Fig. 2:**
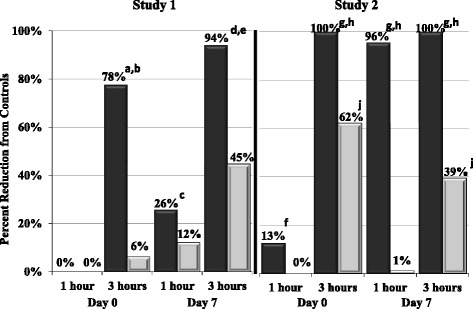
Percent effectiveness, 1 and 3 h post treatment (Day 0) and post infestation (Day 7).  Spinosad,  Afoxolaner. Percentages based on geometric mean live flea counts (normal fleas, excluding those showing abnormal movement) of each group compared to an untreated control group. Different from control: ^a^ *p* = 0.0045; ^c^ *p* = 0.0133; ^d^ *p* = 0.0001; ^g^ *p* < 0.0001; ^j^ *p* = 0.0220. Different from afoxolaner: ^b^ *p* = 0.0059; ^e^ *p* = 0.0009; ^f^ *p* = 0.0276; ^h^ *p* < 0.0001

In both studies arithmetic and geometric mean live flea counts (fleas showing normal movement, plus moribund fleas) in the spinosad groups were significantly lower than in the control groups at all but the first assessment (completed 1 h post treatment) (Tables 3 and 4). Compared to the afoxolaner group, the percent effectiveness of spinosad was significantly greater in both studies at 3 h post treatment and at 1 and 3 h after the Day 7 infestation, and at 1 h post treatment in Study 2 (Fig. 1). When moribund fleas were excluded from counts (i.e., counted as dead), most of the between-group statistical differences are retained (Fig. 2) (Tables 5 and 6).

**Table 3:** Study 1: mean (standard error) (SE) live flea counts^a^ of control, afoxolaner and spinosad group dogs

	Mean	Control	Afoxolaner	Spinosad
Day 0 - 1 hour	Geometric	82.5 (0.1)	90.6 (0.1)	85.1 (0.1)
	Arithmetic	83.7 (3.8)	90.6 (3.8)	85.2 (3.8)
Day 0 - 3 hours	Geometric	81.8 (0.2)	79.4 (0.2)	38.5 (0.2)^b,c^
	Arithmetic	82.3 (6.7)	80.2 (6.7)	44.7 (6.7)^b,c^
Day 7 - 1 hour	Geometric	95.3 (0.1)	83.7 (0.1)	75.2 (0.1)^d^
	Arithmetic	95.5 (4.2)	84.0 (4.2)	76.5 (4.2)^d^
Day 7 - 3 hours	Geometric	89.2 (0.4)	67.4 (0.4)	12.4 (0.4)^e,f^
	Arithmetic	89.7 (4.9)	67.8 (4.9)^e^	21.0 (4.9)^e,f^

^a^Includes moribund fleas, counted as live

Treated group different from control: ^b^ *p* ≤ 0.0051; ^d^ *p* ≤ 0.0128; ^e^ *p* = 0.0069

Different from afoxolaner: ^c^ *p* ≤ 0.0065; ^f^ *p* ≤ 0.0054

**Table 4:** Study 2: mean (standard error) (SE) live flea counts^a^ of control, afoxolaner and spinosad group dogs

	Mean	Control	Afoxolaner	Spinosad
Day 0 - 1 hour	Geometric	81.2 (0.1)	86.0 (0.1)	71.0 (0.1)^b^
	Arithmetic	81.8 (4.2)	86.3 (4.2)	71.8 (4.2)^b^
Day 0 - 3 hours	Geometric	89.8 (0.5)	66.3 (0.5)	6.4 (0.5)^c,d^
	Arithmetic	90.0 (7.3)	70.8 (7.3)	16.5 (7.3)^c,d^
Day 7 - 1 hour	Geometric	91.2 (0.1)	88.9 (0.1)	38.1 (0.1)^c,d^
	Arithmetic	91.5 (4.5)	89.2 (4.5)	41.5 (4.5)^c,d^
Day 7 - 3 hours	Geometric	89.8 (0.6)	69.0 (0.6)	4.8 (0.6)^c,d^
	Arithmetic	90.0 (5.4)	70.0 (5.4)^e^	16.2 (5.4)^c,d^

^a^Includes moribund fleas, counted as live

Treated group different from control: ^c^ *p* ≤ 0.0007; ^e^ *p* = 0.0188

Different from afoxolaner: ^b^ *p* ≤ 0.0276; ^d^ *p* ≤ 0.0019

**Table 5:** Study 1: mean (standard error) (SE) live flea counts^a^ of control, afoxolaner and spinosad group dogs

	Mean	Control	Afoxolaner	Spinosad
Day 0 - 1 hour	Geometric	81.0 (0.1)	90.6 (0.1)	85.1 (0.1)
	Arithmetic	82.7 (4.3)	90.8 (4.3)	85.2 (4.3)
Day 0 - 3 hours	Geometric	81.8 (0.3)	76.7 (0.3)	18.4 (0.3)^b,c^
	Arithmetic	82.3 (6.1)	77.7 (6.1)	27.8 (6.1)^b,c^
Day 7 - 1 hour	Geometric	95.3 (0.1)	83.7 (0.1)	70.8 (0.1)^d^
	Arithmetic	95.5 (4.2)	84.0 (4.7)	72.8 (4.7)^d^
Day 7 - 3 hours	Geometric	88.8 (0.4)	49.1 (0.4)	5.4 (0.4)^e,f^
	Arithmetic	89.3 (6.6)	57.7 (6.6)^g^	8.5 (6.6)^e,f^

^a^Counts of live fleas with normal movement; excludes those with abnormal movement, classified as moribund

Treated group different from control: ^b^ *p* ≤ 0.0045; ^d^ *p* ≤ 0.0133;^e^ *p* ≤ 0.0001; ^g^ *p* = 0.0039

Different from afoxolaner: ^c^ *p* ≤ 0.0001; ^f^ *p* ≤ 0.0054

**Table 6:** Study 2: mean (standard error) (SE) live flea counts^a^ of control, afoxolaner and spinosad group dogs

	Mean	Control	Afoxolaner	Spinosad
Day 0 - 1 hour	Geometric	81.2 (0.1)	86.0 (0.1)	71.0 (0.1)
	Arithmetic	81.8 (4.2)	86.3 (4.2)	71.8 (4.2)^b^
Day 0 - 3 hours	Geometric	89.8 (0.3)	33.9 (0.3)^c^	0.0 (0.0)^d,e^
	Arithmetic	90.0 (9.6)	51.2 (9.6)^c^	0.0 (0.0)^d,e^
Day 7 - 1 hour	Geometric	89.9 (0.4)	88.9 (0.4)	4.0 (0.4)^d,e^
	Arithmetic	90.3 (3.9)	89.2 (3.9)	9.5 (3.9)^d,e^
Day 7 - 3 hours	Geometric	89.8 (0.1)	54.3 (0.1)^f^	0.0 (0.6)^d,e^
	Arithmetic	89.8 (0.1)	56.7 (4.1)^f^	0.0 (0.0)^d,e^

^a^Counts of live fleas with normal movement; excludes those with abnormal movement, classified as moribund

Treated group different from control: ^c^ *p* ≤ 0.0220; ^d^ *p* < 0.0001; ^f^ *p* ≤ 0.0006

Different from afoxolaner: ^b^ *p* = 0.0276; ^e^ *p* < 0.0001

Afoxolaner comparisons to untreated controls, based on geometric live flea counts, failed to achieve statistically significant reductions at all assessments. When arithmetic mean live flea counts were calculated, the only statistically significant differences between the afoxolaner and control groups were on Day 7 at 3 h post infestation (Tables 3 and 4). At this time, arithmetic mean reductions relative to controls were 22.2 % (*p* = 0.0188) and 24.4 % (*p* = 0.0069). When moribund fleas were excluded from live flea counts, afoxolaner group geometric mean counts were significantly lower than in the control group 3 h after treatment on Day 0, when efficacy was 62.2 % (*p* = 0.0220) (Study 2) (Table 6; Fig. 2). There were significant afoxolaner group reductions in arithmetic means of 35.5 % (*p* = 0.0039) and 37.0 % (*p* < 0.0001), in studies 1 and 2, respectively.

## Discussion

These studies demonstrated that spinosad produces a significantly faster flea knockdown than afoxolaner, and that at the end of the month the effectiveness of spinosad is maintained. The results confirm earlier laboratory work in pivotal dose confirmation studies for registration by the FDA, demonstrating that by killing fleas before they can lay eggs, spinosad has the potential to break the flea life cycle when used according to label. This demonstration of the rapid onset of spinosad’s flea-killing activity continues to align it with nitenpyram in quickly eliminating flea infestations from infested dogs, and in maintaining a superior speed of action to afoxolaner for at least one week after treatment. In assessing the relevance of these laboratory studies to other reported studies with spinosad, some considerations are relevant.

First, in each of the studies reported in this paper, the fleas used to infest dogs were laboratory-reared from each research facility’s flea colonies which are regularly refreshed with locally sourced wild-type fleas. This is in contrast to other reported studies in which long-established laboratory strains are selected and used repeatedly to demonstrate a lack of effectiveness that appears to be at variance with real world findings.

Second, those laboratory studies conflict with real-world evidence. For instance, two reports of such experimental studies found that fipronil and selamectin were significantly more effective than spinosad [12, 15]. Yet, when spinosad was compared to these products in field studies in Europe and the United States, not only was spinosad’s effectiveness remarkably high following each of two or three monthly treatments, but it was significantly more effective than either of these topically applied products [5–7]. In those field studies spinosad also produced a better result in alleviating pruritus and other signs of flea allergy dermatitis. It is therefore clear that the laboratory-derived suggestion that the reported topically applied products were more effective than spinosad is soundly contradicted by field data that demonstrate the reverse. Moreover, the concept of spinosad variability arising from laboratory studies conflicts with the consistency spinosad has shown in field studies. In fact, spinosad appears to be unique among flea control products in showing >99 % effectiveness in a number of separate multi-clinic studies across Europe and the United States in which the product was administered monthly by dog owners [5–10].

Thus, while laboratory studies provide a useful means of assessing a product’s potential utility, they may not be reflective of real world results and the ultimate test of any product is how well it performs in the hands of the veterinary client.

## Conclusion

Two laboratory studies demonstrated a superior speed of action of orally administered spinosad compared to afoxolaner at 1 and 3 h post treatment of existing flea infestations, and after a flea challenge at seven days post treatment. A separate study demonstrated that spinosad continues to maintain a high level of residual flea kill through four weeks after treatment. Taken together, these results align with findings from field studies in which monthly treatments with spinosad have provided consistent flea control, along with associated benefits of alleviation in flea-related pruritus.

## References

[1] Dryden MS, Magid-Denenberg T, Bunch S, Boyer J, Schenker R (2001). Control of fleas on dogs and cats and in homes with the combination of oral lufenuron and nitenpyram. Vet Ther.

[2] Dryden MW, McCoy CM, Payne PA (2001). Speed of flea kill with nitenpyram tablets compared to imidacloprid spot on and fipronil spot on in dogs. SupplCompendContinEducPract Vet.

[3] McCoy C, Broce AB, Dryden MW (2008). Flea blood feeding patterns in cats treated with oral nitenpyram and the topical insecticides imidacloprid, fipronil and selamectin. Vet Parasitol.

[4] Blagburn BL, Young DR, Moran C, Meyer JA, Leigh-Heffron A, Paarlberg T (2010). Effects of orally administered spinosad (Comfortis) in dogs on adult and immature stages of the cat flea (*Ctenocephalidesfelis*). Vet Parasitol.

[5] Robertson-Plouch C, Baker KA, Hozak RR, Zimmermann AG, Parks SC, Herr C (2008). Clinical field study of the safety and efficacy of spinosad chewable tablets for controlling fleas on dogs. Vet Ther.

[6] Wolken S, Franc M, Bouhsira E, Wiseman S, Hayes B, Schnitzler B (2012). Evaluation of spinosad for the oral treatment and control of flea infestations on dogs in Europe. Vet Rec.

[7] Dryden MW, Ryan WG, Bell M, Rumschlag AJ, Young LM, Snyder DE (2013). Assessment of owner-administered monthly treatments with oral spinosad or topical spot-on fipronil/(S)-methoprene in controlling fleas and associated pruritus in dogs. Vet Parasitol.

[8] Freedom of Information Summary. Original New Animal Drug Application (NADA 141-406). NEXGARD Afoxolaner Chewable Tablet Dogs. Date of Approval: September 14, 2013.

[9] Meadows C, Guerino F, Sun F (2014). A randomized, blinded, controlled USA field study to assess the use of fluralaner tablets in controlling canine flea infestations. Parasit Vectors.

[10] Saridomichelakis MN, Chatzis MK, Petanides T, Papadopoulos E (2015). A field trial of spinosad for the treatment and prevention of flea infestation in shepherd dogs living in close proximity to flea-infested sheep. Parasit Vectors.

[11] Dryden MW, Payne PA, Vicki S, Kobuszewki D (2011). Efficacy of topically applied dinotefuran formulations and orally administered spinosad tablets against the KS1 flea strain infesting dogs. J Appl Res Vet Med.

[12] Dryden MW, Payne PA, Smith V, Berg TC, Lane M. Efficacy of selamectin, spinosad, and spinosad/milbemycin oxime against the KS1 *Ctenocephalidesfelis*flea strain infesting dogs. Parasit Vectors. 2013. doi:10.1186/1756-3305-6-80. 10.1186/1756-3305-6-80 PMC3621083 23531322

[13] Ross DH, Arther RG, von Simson C, Doyle V, Dryden MW. Evaluation of the efficacy of topically administered imidacloprid + pyriproxyfen and orally administered spinosad against cat fleas (*Ctenocephalidesfelis*): impact of treated dogs on flea life stages in a simulated home environment. Parasit Vectors. 2012. doi:10.1186/1756-3305-5-192. 10.1186/1756-3305-5-192 PMC3514229 22958307

[14] Varloud M, Fourie JJ, Blagburn BL, Deflandre A. Expellency, anti-feeding and speed of kill of a dinotefuran-permethrin-pyriproxyfen spot-on (Vectra®3D) in dogs weekly challenged with adult fleas (*Ctenocephalidesfelis*) for 1 month-comparison to a spinosad tablet (Comfortis®). Parasitol Res. 2015. doi:10.1007/s00436-015-4470-7. 10.1007/s00436-015-4470-7 PMC4478438 25869961

[15] Beugnet F, Doyle V, Murray M, Chalvet-Monfray K (2011). Comparative efficacy on dogs of a single topical treatment with the pioneer fipronil/(S)-methoprene and an oral treatment with spinosad against *Ctenocephalidesfelis*. Parasite.

